# Reverse mentoring to enhance research integrity climate

**DOI:** 10.1186/s13104-022-06098-w

**Published:** 2022-06-17

**Authors:** Daniel Pizzolato, Kris Dierickx

**Affiliations:** Department of Public Health and Primary Care, Centre for Biomedical Ethics and Law, KU Leuven, 3000 Louvain, Belgium

**Keywords:** Reverse mentoring, Mentoring, Learning environment, Research integrity, Research integrity climate

## Abstract

Mentors have the responsibility to guide their mentees through academic and scientific challenges that they might encounter during their educational and professional development. In embodying the role of mentors, senior academics are also expected to transmit knowledge and competencies on the topic of research integrity to their junior colleagues. However, senior academics do not always succeed in transmitting responsible research practices and enhancing the research integrity climate. The implementation of the concept of reverse mentoring can be an option to overcome this issue. Different from traditional mentoring, the flow of information is reversed, going from juniors to seniors. Reverse mentoring, as a developmental partnership between mentees and mentors, has been already used successfully within the private sector and in medical education. In times in which most universities invest resources in organizing dedicated research integrity trainings for PhD candidates and junior researchers, it would be valuable to consider reverse mentoring for fostering responsible research practices and enhancing the research integrity climate. PhD candidates and junior researchers can join and fully contribute to the endeavor of enhancing the research integrity climate by co-creating, together with their senior colleagues a new-shared learning environment.

## Introduction

Universities and research institutions rely heavily on senior academics. Besides having the central role of facilitating the development of research skills and competencies, senior academics have the role of transmitting professional values and, promoting high research integrity standards at the individual and the collective level. Research integrity is defined as performing research according to responsible research practices, in line with high professional, methodological and ethical standards [1]. Universities rely on senior researchers and professors to be knowledgeable and skilled concerning all the issues on the topic of research integrity. In addition, in acting as mentors, senior academics are expected to transfer to junior researchers and PhD candidates all their competencies in terms of research integrity and responsible research practices. As mentors, they should be able to transmit professional values and implicitly act as role models [2–4].

Although senior academics are expected to be in charge of the process of socialization, they do not always succeed in the task, falling short in creating, fostering, and maintaining a healthy research integrity environment [5]. In other words, senior academics sometimes miss the opportunity to transfer research integrity competencies and responsible research practices to their junior counterparts. This can be due to their lacking attention toward research integrity practices or being busy with academic and administrative impediments. Deficiency in providing research integrity training to junior colleagues might endanger the preservation of a healthy and responsible research environment [3, 6]. A previous study highlights that junior researchers and PhD candidates are more aware and skilled than senior colleagues in research integrity [7]. Similarly, a recent study has shown how PhD candidates perceived the surrounded environment as not promoting scientific integrity [8]. A solution to overcome this problem is by implementing the concept of reverse mentoring within the academia. Reverse mentoring is formally defined as the pairing of a younger, junior employee acting as the mentor to share expertise with an older, senior colleague as the mentee [9]. The normal flow of information, knowledge, skills, support, and awareness that normally goes from mentors to mentees is reversed.

## Main text

The concept of reverse mentoring has been successfully implemented within different corporations and private settings since it was first formally introduced at General Electric in 1999 [9–13]. At first, junior employees (acting as mentors) were matched with more senior colleagues (acting as mentees) to transfer specific competencies in new emerging technologies and IT skills. Besides transmitting IT skills, reverse mentoring has been proven to be beneficial in terms of building cross-generational relationships, creating equity and equality within the working environment, creating a two-way flow of new competencies, awareness, skills and establishing a better understanding of the organizational environment [9, 11–15]. Reverse mentoring has also been successfully implemented in the NHS (National Health Service, https://www.nhs.uk/), focusing on exploring equality, diversity, and inclusion by pairing senior white leaders (considered as mentees in the program) with black and minority staff (considered as mentors in the program) [16].

However, reverse mentoring has also disadvantages that lie in its structure. These disadvantages may be related to the level of confidence of junior colleagues when acting as mentors, the willingness of seniors to accept and be responsive to what they are learning when acting as mentees, and issues concerning cross-generational differences [9, 17, 18].

Without being limited to the experiences in the private sector and in the NHS, it is fair to explore whether reverse mentoring might benefit the academic environment [19]. In the context of education, reverse mentoring has been defined as a ‘developmental partnership between one or more less experienced mentor/s (e.g., Ph.D. candidate, junior researcher) providing specific expertise and one or more experienced mentee/s (e.g., supervisor, research team leader, senior professor) who want/s to gain this knowledge/skills’. The partnership is characterized by reciprocity and mutual respect and it aims at both, the development of the mentors and the mentees [20].

Besides being possibly beneficial to the research environment in its entirety, reverse mentoring might be useful to foster responsible research practices and enhance the research integrity climate in particular. Although senior academics can contribute to creating a responsible research environment by fostering research integrity awareness and practices, it might be the right time to let junior researchers join this endeavor. Universities are already investing resources in their PhD candidates and undergraduates by providing dedicated training sessions on research integrity that rarely target senior academics [21–23]. Alongside promoting research integrity, reverse mentoring can be implemented also in relation to open science practices (e.g., open access publications, pre-registration of study designs, pre-print publications and making research materials freely available), aiming to increase transparency and accessibility within the research setting [24]. Junior researchers are often more skilled and better informed than their senior colleagues in relation to open science practices [25, 26]. For this reason, they might be in the position to influence the research environment toward open science practices [25]. The European Commission is also investing in training for PhD candidates, undergraduates and junior researchers by funding different projects focusing on research integrity education such as Path2Integrity [27] and Integrity [28], and open science practices such as Foster [29] and Diosi [30].

**Fig. 1:**
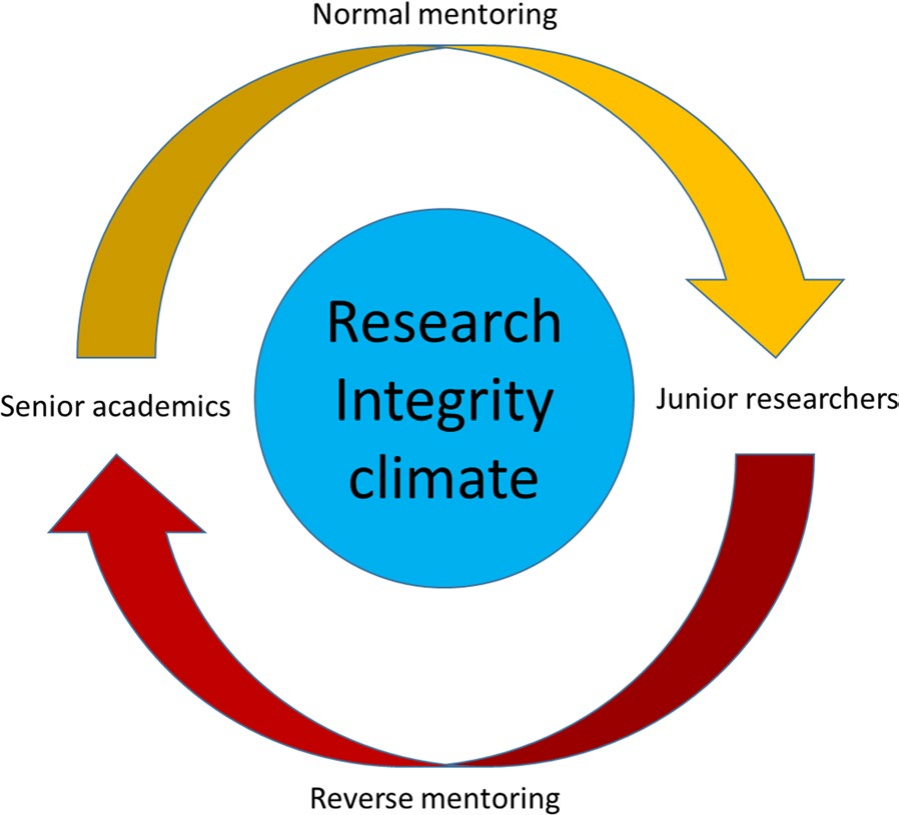
Junior and senior academics collaboration

According to the reverse mentoring model, doctoral candidates and junior researchers (acting temporarily as mentors) can provide seniors (acting temporarily as mentees) with a new impulse in terms of awareness and more up-to-date knowledge on the topic of research integrity and open science. Junior researchers can start collaborating with senior colleagues in giving new momentum to research integrity and a start to the discussion surrounding open science practices within the research environment. This collaboration can only be extremely beneficial to enhancing the research integrity climate (Fig. 1).

PhD candidates and junior researchers can assist senior academics in fostering research integrity in different possible ways. First, junior researchers can collaborate with senior colleagues in discussing and promoting activities in relation to research integrity. Second, junior researchers can provide monthly seminars on general or more discipline-specific research integrity issues within their research teams. Third, junior researchers can update new colleagues (junior and senior) on what is expected from them in terms of research integrity, responsible research practices and open science. Forth, junior researchers with specific expertise on the topic of research integrity and open science can facilitate workshops involving senior colleagues. Finally, junior researchers can be involved in the definition of some recommendations to regulate the supervisor–supervisee relationship. Besides having possible direct benefits in fostering responsible research practices and in promoting open science practices, reverse mentoring might have an impact on issues not strictly related to research integrity or open science, but still influencing the research integrity climate. Reverse mentoring might be valuable for senior academics for improving their mentorship and inter-relational skills and for junior researchers to improve their leadership and managerial skills [10–14]. Supporting reverse mentoring and the collaboration between junior researchers and senior academics might be beneficial to mitigating the intergenerational gap between them and the power imbalance that occasionally suffocates early-career researchers and PhD candidates.

### Outlook

Overcoming the issue of senior academics’ occasional inability to foster responsible research practices and to enhance the research integrity climate has to be taken seriously. Universities should start recognizing and supporting the role that PhD candidates and junior researchers play in fostering research integrity. Universities should start involving PhD candidates and junior researchers in the organization of and in facilitating training sessions on research integrity and open science. Involving juniors in the discussion surrounding responsible conduct of research and practices could provide some much-needed momentum to the topic of research integrity. Junior researchers and doctoral students should start joining senior colleagues in enhancing the research integrity climate by creating a shared learning environment.

## Data Availability

Not applicable.

## Abbreviation

NHS: National Health Service
